# The relationship between dental anxiety and oral health literacy with oral health-related quality of life

**DOI:** 10.1186/s12903-024-04359-7

**Published:** 2024-05-14

**Authors:** Mohammad Samami, Hassan Farrahi, Mahsa Alinia

**Affiliations:** 1 Dental Sciences Research Center, Department of Oral and Maxillofacial Medicine, School of Dentistry, Guilan University of Medical Sciences, Rasht, Iran; 2 Kavosh Cognitive Behavior Sciences and Addiction Research Center, Department of Psychiatry, School of Medicine, Guilan University of Medical Sciences, Rasht, Iran; 3 General dentist, Rasht, Iran

**Keywords:** Dental anxiety, Health literacy, Quality of life

## Abstract

**Background and Aim:**

Dental anxiety is a prevalent issue in society, characterized by an uneasy sensation and anticipation of negative experiences in dental settings. In essence, dental anxiety, oral health literacy, and quality of life may have a relationship with each other, however, there is a shortage of evidence examining the interplay between these factors. Therefore, this study aimed to assess the relationship between dental anxiety and oral health literacy (OHL) with oral health-related quality of life (OHRQOL).

**Methods:**

This is an analytical cross-sectional study conducted on 155 patients referred to the Department of Oromaxillofacial Diseases. Three questionnaires consisting of dental anxiety scale, oral health impact profile- 14, and oral health literacy adult questionnaire were used to measure anxiety, health literacy, and the quality of life-related to oral health. Scores were recorded and analyzed by IBM SPSS 24 software using independent samples T-test and ANOVA. Besides, the confirmatory modeling through the goodness of fit index of the model was applied.

**Results:**

This study involved 155 participants, with a mean age of 38.44 ± 14 years. The majority were females, comprising 99 individuals (63.9%). In this study, 89 patients (57.4%) had dental anxiety. The mean OHL score in the examined participants was 9.88 ± 3.97. Both factors of anxiety (*p* < 0.001) and OHL (*p* = 0.012) had a significant effect on the OHRQOL. There was no significant difference in the mean OHRQOL among the three categories of OHL (*p* = 0.085). The confirmatory modeling showed that only the fourth (*p* = 0.065) and fifth (*p* = 0.146) questions of the OHL questionnaire had no significant effect on the total score of OHL. Besides, both factors of anxiety (*p* < 0.001) and OHL (*p* = 0.012) had a significant effect on OHRQOL. With an increase of one unit in anxiety, the OHRQOL score increases by 0.31 and for a one-unit increase in the OHL score, the OHRQOL score decreases by 0.66 units.

**Conclusion:**

In conclusion, it seems that considering various dimensions of oral and dental health can help patients to have reduced psychological anxiety. Notably, further multicenter studies assessing diverse variables related to dental anxiety, OHL, and OHRQOL, and considering more comprehensive study designs with longitudinal follow-up could help provide insights into how changes in dental anxiety and OHL over time affect OHRQOL.

## Introduction and aims

Dental anxiety is a prevalent issue in society, characterized by an uneasy sensation and anticipation of negative experiences in dental settings [1–3]. This anxiety is widespread, affecting a significant portion of the population at both individual and community levels [4]. It is observed across genders and age groups [5], though it tends to be more prevalent among women [6–8] and younger adults [9–11].

Over a decade, oral health literacy (OHL) has gathered significant interest from healthcare professionals and policymakers due to its considerable effect on oral health outcomes [12, 13]. Considered a multifaceted concept, OHL has consistently been examined in dental research [13, 14] and is indicated as a potential determinant of oral health behaviors, outcomes, and access to dental services [14].

In essence, both dental anxiety and OHL affect oral and dental health. Conversely, oral and dental well-being significantly impacts general health and quality of life (QOL) by affecting oral and dental function as well as social interactions. Oral health-related quality of life (OHRQOL) serves as a measure for assessing the impact of oral and dental health on individuals’ overall well-being [15]. OHRQOL can be pivotal at the population level in driving a country’s development, prosperity, and social advancement. Thus, it is imperative to identify the factors associated with it to devise and implement necessary strategies aimed at enhancing oral health.

Although there is a high prevalence of anxiety disorders, there is a shortage of evidence and gap in the literature regarding studies examining the interplay between dental anxiety, OHRQOL, and OHL. Therefore, gaining a deeper understanding of the relationship between these factors is essential for enhancing oral health outcomes, improving quality of life, and reducing individual treatment expenses and this study aimed to assess this issue. Implementing such a study at the community level is imperative not only for enhancing physical and mental well-being but also for reducing preventable costs and guiding treatment policies across various disciplines.

## Methods

### Participants and settings

This analytical cross-sectional study was conducted on 155, 18-80-year-old patients (63.9% females and 36.1% males) who were admitted to the dental school of Guilan University of Medical Sciences in 2023. The study utilized consecutive sampling based on daily patient visits to the Department oromaxillofacial Diseases at School of Dentistry, Iran. Inclusion criteria comprised individuals aged 18 to 80, proficient in Persian language, without mental disorders, mental retardation, or acute psychiatric conditions such as psychosis under treatment. Smokers and those with systemic diseases, deafness, or blindness were excluded.

### Data gathering

First, the people were asked whether they had a history of visiting a psychiatrist or psychologist, or whether they had received a special drug concerning their mental discomfort or not. Personal information including age, gender, education level, income status, and place of residence (urban or rural) by the patient was registered. Also, the participants were assessed for three factors, including dental anxiety, level of OHL, and OHRQOL.

### Modified dental anxiety scale (MDAS)

The Modified Dental Anxiety Scale (MDAS) is designed to measure dental anxiety and comprises five questions with five-choice responses. These questions assess a patient’s anxiety levels in various dental scenarios: waiting for an appointment in a dental clinic, awaiting treatment in a dental office, waiting in the dental chair for teeth grinding, waiting in the dental chair for scaling, and waiting in the dental chair for local anesthesia injection. Each question offers five potential responses, ranging from no anxiety (score 1) to severe anxiety (score 5). The questionnaire’s score range is from 5 to 25. Patients scoring 11 or higher are categorized as having dental anxiety, while a score of 19 or higher indicates significant levels of dental anxiety warranting special attention. The Persian version of this questionnaire’s validity and reliability were confirmed in the study by Saatchi et al. [16].

### Adult oral health literacy questionnaire (OHL-AQ)

The Adult OHL Questionnaire (OHL-AQ) was utilized to assess the OHL of participants. This questionnaire, tailored for the Iranian context and validated for reliability [17], comprises 17 questions divided into 4 sections. It evaluates, reading comprehension, numeracy, listening, and decision-making regarding oral health issues. Correct answers received 1 point, while incorrect or unanswered questions received 0 points. Participants’ total correct answers range from 0 to 17. OHL scores are categorized into three groups: insufficient (scores 0–9), borderline (scores 10–11), and sufficient [12–17]. The Short Test of Functional Health Literacy in Adults delineates scores into inadequate (0–53), borderline (54–66), and sufficient (67–100) categories [17].

### Oral health-related quality of life (OHRQOL)

This questionnaire comprises 14 questions designed to evaluate the impact of oral health across seven dimensions: functional limitations, physical discomfort, psychosocial distress, physical impairment, social limitations, and perceived shortcomings. It aims to identify all conditions associated with individuals’ QOL and the extent to which they have experienced these effects over the past six months. Responses are rated as follows: often = 4, frequently = 3, sometimes = 2, rarely = 1, and never = 0. The index scores for oral health effects range from 0 (indicating no adverse effects in the past month) to 56 (frequent experience of all adverse effects in the past month). Therefore, lower scores meant better quality of life. The questionnaire has been translated into Persian by Motallebnejad et al. and its reliability and validity have been confirmed [18].

### Ethical considerations

The written informed consent letter was obtained from patients. This study was approved by the Ethics Committee of the Vice Chancellor of Research at Guilan University of Medical Sciences (code: IR.GUMS.REC.1402.135, approval Date: 2023-05-31).

### Statistical analysis

Qualitative data were described using numbers and percentages, while quantitative data were summarized using mean and standard deviation. To analyze the research hypotheses, assumptions related to parametric tests were initially assessed by the Kolmogorov- Smirnov test. Normally-distributed quantitative variables were compared by the independent sample T-test. We compared three categories of OHL based on HRQOL by the ANOVA test. To determine the relationship between dental anxiety and the level of OHL with the QOL related to oral health in patients, confirmatory modeling was used. After fitting the model, the goodness of fit index of the model was examined. A goodness-of-fit index, in structural equations modeling meant an index for assessing the fit of a model to data that ranges in possible value between zero and one. In this case, zero indicates a complete lack of fit and one shows perfect fit. These analyses were conducted using IBM SPSS statistics software version 24, with a significance level set at 0.05 for all tests.

## Results

This study involved 155 participants, with a mean age of 38.44 ± 14 years. The majority were females, comprising 99 individuals (63.9%). Among the educational levels, a bachelor’s degree was the most common, with 55 participants (35.5%), followed by diploma holders (50 participants, 32.3%), and Ph.D. holders (20 participants, 12.9%). Urban residents accounted for 134 individuals (86.5%), while the remainder resided in rural areas. Regarding income distribution, 60 participants (38.7%) reported high income, 51 (32.9%) reported low income and 44 (28.4%) reported middle income.

The mean OHRQOL among the participants was 18.26 ± 12.13. Dental anxiety was present in 89 individuals (57.4%), with a mean score of 12.18 ± 5.37. The majority demonstrated insufficient OHL (77 participants, 49.7%), followed by borderline (20 participants, 12.9%), and adequate literacy (58 participants, 37.4%). The mean OHL score was 9.88 ± 3.97.

Table 1 illustrates that the mean OHRQOL significantly differed between individuals without dental anxiety and those with dental anxiety (*p* = 0.006). Specifically, individuals with dental anxiety exhibited higher average scores (worse condition) in OHRQOL. However, there was no significant difference in the mean OHRQOL among the three categories of OHL (*p* = 0.085).

**Table 1:** The relationship between dental anxiety level and health literacy with quality of life-related to oral health

	Quality of life	*p*-value
	Mean ± SD	
Dental Anxiety		
Without dental anxiety	15.18 ± 10.58	0.006 Independent Samples T-Test
With dental anxiety	20.55 ± 12.75	
Oral health literacy		
Insufficient	20.22 ± 13.20	0.085 ANOVA
Borderline	18.60 ± 12.44	
Sufficient	15.55 ± 10.06	

Table 2 illustrates significant variations in the mean QOL across different age groups and categories of dental anxiety. Specifically, among individuals experiencing dental anxiety, those aged over 38 exhibited a higher average score of OHRQOL (*p* = 0.022). Moreover, among individuals with dental anxiety, women showed significantly higher OHRQOL (*p* < 0.001). Notably, significant differences in average QOL were observed among individuals with diplomas (*p* < 0.001) in the context of dental anxiety. Furthermore, urban (*p* = 0.031) and rural (*p* = 0.019) residents with dental anxiety displayed notably distinct average quality of life scores related to oral health, with higher scores observed in both settings among those experiencing dental anxiety. Individuals with low income exhibited a higher average OHRQOL in the dental anxiety category (*p* = 0.011). In contrast, there were no significant differences in the average OHRQOL across the three categories of OHL among individuals aged less than 38 years (*p* = 0.742) and those aged 38 years or older (*p* = 0.132). Similarly, no significant differences were observed in average QOL among men (*p* = 0.448) and women (*p* = 0.107) across the three categories of OHL. Additionally, no significant differences were found among individuals with diplomas (*p* = 0.730) and bachelor’s degrees (*p* = 0.528) across the three categories of OHL. However, a significant difference in the average OHRQOL was observed among individuals living in rural areas (*p* = 0.041).

**Table 2:** Comparing the relationship between the level of dental anxiety and the OHRQOL

Variables	Anxiety	Mean ± SD	*P*-value Independent Samples T-Test
Age			
< 38 years	Without dental anxiety	12.59 ± 8.95	0.160
	With dental anxiety	15.85 ± 10.95	
≥ 38 years	Without dental anxiety	17.94 ± 11.58	0.022
	With dental anxiety	24.56 ± 12.90	
Gender			
Male	Without dental anxiety	12/19 ± 16/09	0.759
	With dental anxiety	11/24 ± 15/09	
Female	Without dental anxiety	14.22 ± 8.63	< 0.001
	With dental anxiety	22.34 ± 12.78	
Level of Education			
Guidance school	Without dental anxiety	21.60 ± 11.28	0.150
	With dental anxiety	31.25 ± 3.86	
high school	Without dental anxiety	25.67 ± 4.04	0.109
	With dental anxiety	15.50 ± 6.36	
Diploma	Without dental anxiety	11.12 ± 7.86	< 0.001
	With dental anxiety	25.45 ± 12.57	
Associate degree	Without dental anxiety	27.40 ± 16.95	0.278
	With dental anxiety	16.83 ± 13.47	
Bachelor	Without dental anxiety	15.52 ± 10	0.567
	With dental anxiety	17.87 ± 12.30	
PhD	Without dental anxiety	10.92 ± 6.47	0.618
	With dental anxiety	9.37 ± 6.93	
Place of inhabitants			
City	Without dental anxiety	15.55 ± 11.18	0.031
	With dental anxiety	20.32 ± 13.13	
Village	Without dental anxiety	13.69 ± 7.87	0.019
	With dental anxiety	22.87 ± 8.20	
Income			
Low income	Without dental anxiety	13.78 ± 8.40	0.011
	With dental anxiety	21.70 ± 11.02	
Middle income	Without dental anxiety	14.35 ± 9.76	0.079
	With dental anxiety	19.75 ± 10.02	
High income	Without dental anxiety	16.68 ± 12.40	0.384
	With dental anxiety	19.97 ± 16.10	

Based on the obtained results, only the fourth (preventing dental decays through not using carbohydrates) (*p* = 0.065) and fifth (number of permanent teeth) (*p* = 0.146) questions of the health literacy questionnaire had no significant effect on the total score of OHL. Based on the results, both factors of anxiety (*p* < 0.001) and OHL (*p* = 0.012) had a significant effect on quality of life. With an increase of one unit in anxiety, the OHRQOL score increases by 0.31 and for a one-unit increase in the health literacy score, the OHRQOL score decreases by 0.66 units.

Based on the results obtained in the dental anxiety questionnaire, the second question (the patients’ feelings while waiting in the dental office) had the highest impact on the total anxiety score. In the oral health literacy questionnaire, the seventh question (the schedule for using antibiotics) had the highest impact on the total health literacy score. In the QOL questionnaire, the ninth question (discomfort due to the mouth, teeth, or denture) had the highest impact on the total QOL score. Dental anxiety has a greater effect than oral health literacy on the OHRQOL.

**Fig. 1:**
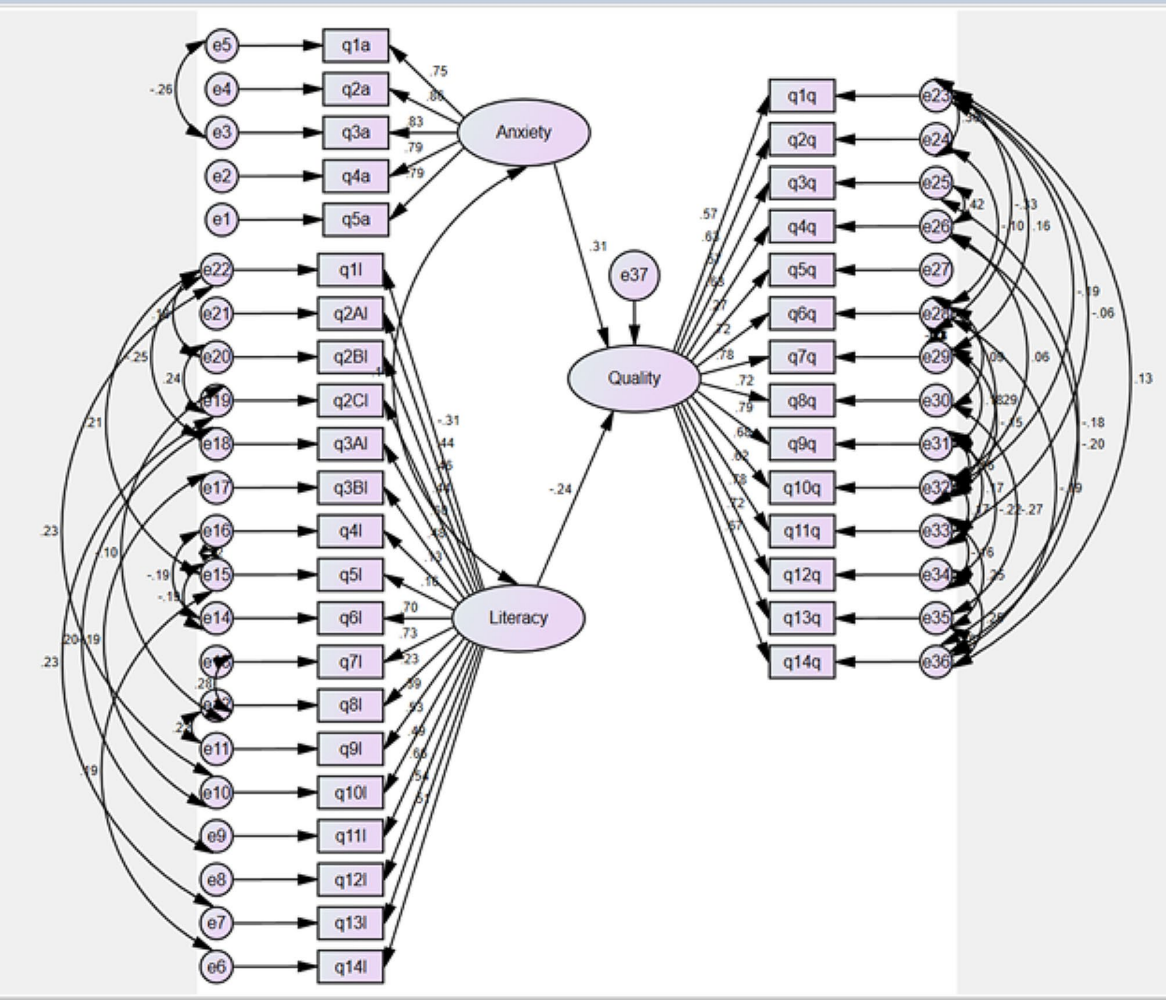
The confirmatory model of external factors assessing the relationship between dental anxiety and the level of oral health literacy with the quality of life

To determine the relationship between dental anxiety and the level of OHL with the OHRQOL in patients, confirmatory modeling through the goodness of fit index of the model showed that only the fourth (preventing dental decays through not using carbohydrates) and fifth (number of permanent teeth) (*p* = 0.146) questions of the health literacy questionnaire had no significant effect on the total score of OHL. Besides, both factors of anxiety (*p* < 0.001) and health literacy (*p* = 0.012) had a significant effect on quality of life. With an increase of one unit in anxiety, the OHRQOL score increases by 0.31 and for a one-unit increase in the health literacy score, the OHRQOL score decreases by 0.66 units. Based on the results obtained in the dental anxiety questionnaire, the second question (the patients’ feelings while waiting in the dental office) had the highest impact on the total anxiety score. In the OHL questionnaire, the seventh question (the schedule for using antibiotics) had the highest impact on the total score. In the QOL questionnaire, the ninth question (discomfort due to the mouth, teeth, or denture) had the highest impact on the total QOL score. The model showed that dental anxiety had a greater effect than OHL on the OHRQOL (Fig. 1).

## Discussion

Dental anxiety can be an obstacle to dental care and a result of insufficient health of the mouth and teeth of the individual. The current investigation showed that the average score for OHRQOL was 18.26 ± 12.13, with 57.4% reporting dental anxiety. Findings underscore the tendency for individuals with dental anxiety to potentially overlook dental visits and neglect their oral health, ultimately leading to various oral disorders and causing a cycle of anxiety and avoidance of treatment, thereby exacerbating oral hygiene deterioration.

In the current study, participants were stratified into two age groups. In both groups, a significant association between dental anxiety and OHRQOL was observed, highlighting the pervasive impact of dental anxiety across age groups, hindering individuals from seeking dental care. As Aardal et al. noted when treating patients with high dental anxiety, dentists should be aware that factors such as general anxiety could complicate the therapeutic setting [19]. Contrary to Saatchi et al. [16] and FarhadiNasab et al. [20], who found no age-related differences in dental anxiety scores, Humphris et al. [21] and Nascimento et al. [22] reported that younger individuals tend to exhibit higher levels of dental anxiety compared to older individuals.

In this investigation, a significant association was found between dental anxiety and OHRQOL among women, contrasting with the findings among men. Consistent with the current results, Gisler et al. [23] and Saatchi et al. [16] also observed higher levels of dental anxiety in women compared to men. Mental health conditions such as anxiety, stress, depression, and panic disorder are more prevalent among women, and dental anxiety may be linked to these disorders [16, 23]. This observation aligns with the notion that women tend to exhibit higher levels of neuroticism, characterized by a predisposition to experience negative emotional states, including anxiety, compared to men. Furthermore, women are often more expressive of their inner states than men, who may be less inclined to openly express anxiety and fear.

However, certain studies such as a review by SE Guney et al. [24] and Folayan et al. [25] reported no gender-based differences in the relationship between dental anxiety and OHRQOL [24, 25]. Discrepancies in findings could be indicated by variations in gender neuroticism levels across different societies, as well as differences in measurement tools and study designs. Additionally, variations in sample sizes could contribute to the differing outcomes observed in various studies. Further investigation into the influence of gender on dental anxiety and its impact on OHRQOL appears warranted in future studies.

The role of income and socio-economic status in shaping the relationship between dental anxiety and OHRQOL across three income levels is an aspect that has not been explored extensively in existing literature. To the best of our knowledge, no prior study has delved into the influence of income levels on the relationship between dental anxiety and OHRQOL.

Regarding OHL, nearly half of the participants (49.7%) demonstrated an insufficient level in this study, consistent with findings from Navabi et al. [26] among patients in Kerman. This investigation revealed a direct and significant correlation between OHL and OHRQOL, indicating that increased OHL is associated with improved OHRQOL [26]. This aligns with results from studies by Batista et al. [27] and Divaris et al. [28], all of which highlighted significantly lower OHRQOL among individuals with lower levels of OHL [27, 28]. However, Nawabi et al. did not find a significant relationship between OHL and OHRQOL in their study [26].

In this investigation, demographic factors such as age, gender, income, and education level did not affect the relationship between OHL and OHRQOL. Similarly, Navabi et al. [26] found no specific association between demographic factors like occupation, education, and gender with the level of OHL [26]. It appears to be a component of general literacy that can be enhanced through various educational channels, including mass media, irrespective of academic education level.

Contrary to the findings of Vyas et al. [29], this study did not find a significant difference in OHL scores relative to education and socio-economic status. However, the results of Baskaradoss et al. [30] indicated a significant difference in OHL across educational levels, highlighting potential inconsistencies in findings that may stem from varying sampling methodologies [30].

Regarding the relationship between dental anxiety and OHL, this study’s results were consistent with Azodo et al. [31]. They noted that individuals with lower levels of OHL exhibited higher levels of dental anxiety, potentially due to a lack of awareness regarding health-related issues and treatment processes, which can exacerbate anxiety. Conversely, increasing literacy in this domain may foster a more realistic and rational perspective on oral health matters [31].

Limitations and strengths:

The study demonstrates notable strengths including its comprehensive examination of the interplay between dental anxiety, OHL, and OHRQOL. The study provides valuable insights into the complicated dynamics of dental anxiety and oral health, underscoring the need for cautious interpretation in clinical applications and future research. However, there are some limitations. The cross-sectional design limits causal inference, while overlooked confounding factors like access to dental care may impact the findings. This study used self-reported measures and this method may induce response bias and affect the accuracy of the results. Although some important factors and differences were examined in this single- center study, the study lacks depth in elucidating underlying reasons. Therefore, further multicenter studies assessing diverse variables related to dental anxiety, OHL, and OHRQOL, and considering more comprehensive study designs can be recommended. Moreover, there was no longitudinal follow-up, therefore noticing this issue could help provide insights into how changes in dental anxiety and OHL over time affect OHRQOL.

## Conclusion

As it was mentioned, there was a synchronous effect of dental anxiety and OHL on OHRQOL. Therefore, it seems that with efficient policies in the field of public education and considering various dimensions of oral and dental health, as well as providing awareness about procedures and the necessity of various treatments, psychological anxiety of patients can be greatly reduced. This subsequently may lead to an increase in the oral and dental health of individuals as well as society. In addition, it can reduce heavy costs of dental treatments on people and the healthcare system. Therefore, it seems that using these questionnaires in patients could have practical effect on the service they receive and help dentists to manage patients effectively.

## Data Availability

All data generated or analyzed during this study are included in this published article.
